# The Impact of Gestational Diabetes in Pregnancy on the Cardiovascular System of Children at One Year of Age

**DOI:** 10.3390/jcm10245839

**Published:** 2021-12-13

**Authors:** Annette Wacker-Gussmann, Judith Schopen, Jana Engelhard, Christina Sitzberger, Nadine Lienert, Peter Ewert, Alexander Müller, Georg Schmidt, Renate Oberhoffer-Fritz, Silvia Maria Lobmaier

**Affiliations:** 1 Institute of Preventive Pediatrics, Faculty of Sport and Health Sciences, Technical University of Munich, 80992 Munich, Germany; janaengelhard@live.de (J.E.); christina.sitzberger@tum.de (C.S.); nadine.lienert@t-online.de (N.L.); renate.oberhoffer@tum.de (R.O.-F.); 2 German Heart Center, Department of Congenital Heart Disease and Pediatric Cardiology, 80663 Munich, Germany; Schopen@dhm.mhn.de (J.S.); peter.ewert@dhm.mhn.de (P.E.); 3 Department of Adult Cardiology, Klinikum rechts der Isar, Technical University of Munich, 81675 Munich, Germany; alexander.mueller@mytum.de (A.M.); gschmidt@tum.de (G.S.); 4 Department of Obstetrics and Gynecology, Klinikum rechts der Isar, Technical University of Munich, 81675 Munich, Germany; silvia.lobmaier@tum.de

**Keywords:** gestational diabetes, cardiovascular system, phase rectified signal averaging, average acceleration capacity

## Abstract

Gestational diabetes mellitus (GDM) is a common complication in pregnancy. The effect of GDM on the cardiovascular system after birth is still unclear. Between August 2015 and December 2018, 205 pregnant women were included in the prospective controlled observational study. Patients with GDM were assigned to the study group (*n* = 99), whereas (*n* = 107) healthy women served as controls. Postnatal follow up of their offspring was performed at 12 months of age. All included children (*n* = 125) underwent a specific standardized protocol including anthropometric data, such as weight, height, body mass index (BMI), blood pressure (BP) recordings and ultrasound measurements of the abdominal aortic intima-media-thickness (IMT). Furthermore, at least 10 min 3-channel electrocardiogram recording was done to evaluate the autonomic nervous system (ANS) by phase rectified signal averaging. There were no significant differences in anthropometric data between the groups, neither in the blood pressure nor in the intima-media-thickness of the aorta abdominals. However, in the study group, significantly lower average acceleration capacity (AAC) (study group −20.10 ± 3.04 ms, control group −18.87 ± 4.00 ms, *p* = 0.02) was found, indicating ANS activation at one year of age. Further studies are required to determine if these results are persistent and if these findings have long-term effects.

## 1. Introduction

Gestational diabetes mellitus (GDM) is a common complication in pregnancy, affecting around 13% [1] of all pregnant women. The incidence and prevalence will presumably increase further over the next decades, because the percentage of high-risk pregnancies increases and lifestyle problems such as obesity become more prevalent. Pregnant women with gestational diabetes have an increased risk of maternal and fetal morbidity during pregnancy and children can suffer complications such as hypoglycemia or macrosomia. The women themselves may develop long-term risk conditions such as severe obesity and diabetes mellitus (type 2). Therefore, a number of cardiovascular sequelae (disorders of lipid metabolism, high blood pressure, myocardial infarction, stroke, etc.) can occur in these mothers [2].

Moreover, significant weight gain has been observed during the ongoing COVID-19 pandemia. Social distancing, more screen time, and less physical activity have all contributed to increased weight gain and therefore the risk of gestational diabetes in pregnancy has also increased [3]. Adhering to nutritional patterns enriched with plant-derived foods, low glycemic index or Mediterranean diet prior to and during pregnancy is reported to have a protective effect, reducing gestational weight gain and the risk of GDM [4].

The ‘Barker Hypothesis’ [5] postulates a correlation between intrauterine exposure of the fetus to GDM and the development of cardiovascular risk factors and heart diseases later in life. Among others, these risk factors include arterial hypertension and obesity in children [6]. In order to prevent the onset of these diseases, there is an increasing interest in investigating the effects of GDM on the developing cardiovascular system in children.

The autonomic nervous system might be a key component influencing these physiological processes. It independently regulates heart rate, blood pressure, respiration, digestion, and sexual arousal [7]. Therefore, changes of the ANS automatically effect the cardiovascular system. It can be resembled by heart rate variability (HRV), regulated through sympathetic and vagal influences, as well as by respiratory, baroreflex, and circadian processes [8]. The average deceleration capacity (ADC) and acceleration capacity (AAC) of heart rate indices were established and assessed as indicators of these complex regulations reflecting the autonomic nervous system (ANS) function, in adults and the fetuses. Indeed, our study group already showed that fetal ANS is affected by maternal GDM [9].

However, to the best of our knowledge, there are no established parameters in children.

The aim of this study was to evaluate the influence of GDM on offsprings’ cardiovascular systems with its primary focus on obesity, blood pressure, and ANS.

## 2. Materials and Methods

Pregnant women with GDM were included in a prospective controlled observational study between August 2015 and December 2018. Healthy pregnant women served as controls. All participants were recruited at the tertiary-level teaching University Hospital “Klinikum rechts der Isar” of the Technical University of Munich between 28- and 37-weeks’ gestation. The pregnant women were addressed during their routine examinations. The suitability of this study, with regard to the inclusion and exclusion criteria, was checked in advance on the basis of the existing files [2].

GDM was diagnosed according to the German guidelines of AG-diabetes and DGG 2003 [10].

Inclusion criteria for the mothers were a confirmed diagnosis of GDM, adult age (>18 years), and obtained informed consent. Exclusion criteria were additional cardiovascular or nephropathic diseases, multiple pregnancies, acute illness including infectious diseases and premature labor, a lack of cognitive competence to consent to research, and refusal to consent.

The pregnant women and their fetuses were examined according to a predefined protocol. For this manuscript we only focus on the postnatal data. Postnatal follow up of the children was done at the age of 12 months (Range 11–13 months). All children underwent a check-up including anthropometric data such as weight, height, body mass index (BMI), blood pressure (BP) recordings, and ultrasound measurements of the abdominal aortic intima-media-thickness (IMT).

Furthermore, a 10 min 3-channel electrocardiogram recording was performed to analyze the autonomic nervous system. The extracted raw data were analyzed off-line to obtain the RR intervals. The average acceleration capacity (ACC) of the heart rate was calculated by the phase-rectified signal averaging method (PRSA) published by Schmidt, G, and Lobmaier, SM, et al. [9].

A 10 min 3-channel electrocardiogram was recorded with the medilog AR4plus (Schiller Medizintechnik GmbH, Feldkirchen, Germany) holter monitoring system. During the recording, the children were either sitting, crawling, walking around, or carried by their parents.

For this specific analysis we used the RR intervals. The ECGs were analyzed with Schiller Darwin 2 (beat detection) and then visually inspected and corrected if necessary.

Phase-rectified signal averaging (PRSA) analysis was used to assess HR variability. This specific technique, primarily established in adults and later on in the fetus, permits detection of quasi-periodicities in non-stationary data [11]. The RR intervals were analyzed by PRSA (phase-rectified signal averaging), a method previously described by Bauer et al. [10,11]. The following parameters were used to calculate the AAC: T = 19 heartbeats and anchor points were defined as decreases in RR intervals between 0.60 and 0.95 percent.

The height was measured with the Seca 210 mobile mat for babies and toddlers. During the examination, the children lay on their backs, barefoot, and were measured from head to heel. The weight was measured with the Seca 384 scale while the children were naked except for their diapers. The percentiles were compared to those by Kromeyer-Hauschild et al. [12].

The blood pressure was taken with the Bosoclinicus II mechanical blood pressure device, using the 8–13 cm cuff on the left arm. BP was measured three times within 10 min while the children were either sitting or held in their parents’ arms. Patients who did not tolerate the cuff were excluded from the measurements. Crying and moving is common during this age, so we had to exclude several children. The blood pressure results were compared between the different groups.

### Intima-Media Thickness

Ultrasound examination was done with the GE Healthcare vivid 7. The dimension and the intima media thickness of the abdominal aorta were measured three times independently right above the origin of the renal arteries from the aorta. We strictly followed the predefined criteria. The study was approved by the local IRB (430/17S).

All data were tested for normal distribution. They are presented as means ± standard deviation (SD). Student’s *t*-test for independent samples or Mann–Whitney U test was used for comparison between groups. *p*-Values < 0.05 were considered statistically significant. Statistical analysis was performed using SPSS version 25.0 (IBM Corp., Armonk, NY, USA).

The further analysis of phase-rectified signal averaging was performed by the programming and numeric computing platform Matlab (mathworks.com, accessed on 10 November 2021).

## 3. Results

A cohort of 205 pregnant women was recruited for the prospective observational study. Women with singleton pregnancies affected by GDM were included in the study group (*n* = 99), whereas healthy women served as controls (*n* = 107).

The mean age of the mothers was 33.84 (SD + 4.7) years in the study group and 32.6 (SD + 4.2) years in the control group. The mean gestational age of the newborns at birth was 40.0 (SD + 0.1) weeks in the study group and 39.1 (SD + 1.2) weeks in the control group. Of all women with gestational diabetes, 52 (53%) were treated with insulin and 47 (47%) with dietary advices only. The mean fasting blood glucose value of the study group was 91.83 ± 8.34 mg/dL. The mean values of 1 and 2 h oGTT were 168.61 ± 30.25 mg/dL and 137.42 ± 32.77 mg/dL in the study group. The values of the control group were all within the normal range [2].

The birth weight of the study group was 3348 g (SD + 375) and 3496 g (SD + 379) in the control group.

A total of 125 children (study group *n* = 63; control group *n* = 62) were included in the one-year follow up study; 39% were lost to follow-up. In the study group, 25 (40%) of the toddlers were female and 32 (52%) in the control group.

### 3.1. Anthropometric Data

All anthropometric data are shown in Table 1. The age at follow up was almost similar in both groups. Neither the absolute values nor the percentiles for height, weight, and BMI showed any significant differences between the groups.

### 3.2. Cardiovascular Data

The systolic blood pressure was within normal limits and showed no statistical significance between the study and the control group. The diastolic blood pressure was also not significantly different in both of our groups (Table 2).

The aortic intima-media thickness showed no significant difference between both groups (Table 2).

**Figure 1:**
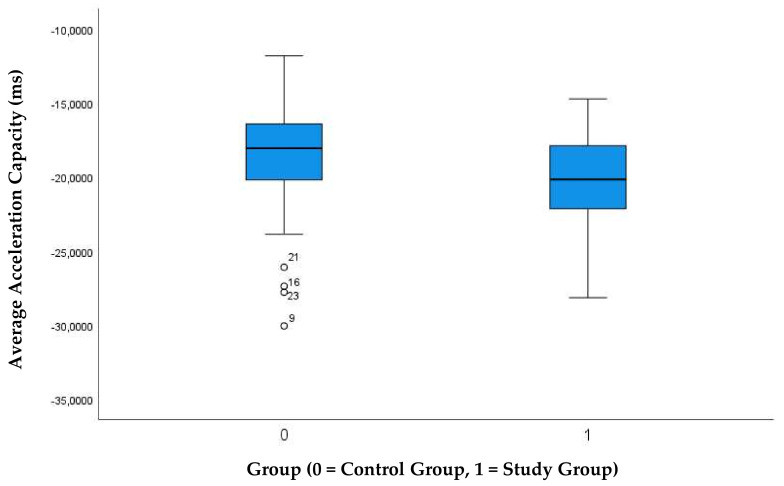
Comparison of the average acceleration capacity between study group and controls.

The autonomous nervous system, elaborated by the phase rectified signal averaging technique, showed an average acceleration capacity which was significantly lower (negative values) in the study group (Table 2, Figure 1).

## 4. Discussion

To the best of our knowledge, the current study reveals for the first time, that the offspring of women with GDM exhibit alterations in the ANS function at one year of age. Other cardiovascular parameters, such as blood pressure or intima-media thickness, did not show any difference between the study group and the control group.

The autonomic nervous system is a component of the peripheral nervous system that regulates involuntary physiologic processes including heart rate, blood pressure, respiration, digestion, and sexual arousal [7]. Therefore, changes of the ANS automatically affect the cardiovascular system.

To reflect the ANS, the fairly new method PRSA was used. In contrast to other methods for the assessment of HR variability that cannot be applied to noisy non-stationary signals, such as those represented by heart rate, the PRSA technique permits changes in complex oscillatory modulations of multiple frequency drivers to be determined [13]. PRSA achieves this not only by calculating the variation of HR, but also by reflecting the speed of changes in HR described as the average acceleration (AAC) and deceleration (ADC) capacities in HR. Their alteration gives established insight into changes in ANS function. PRSA has become a valid method to predict survival after myocardial infarction in adult cardiology [14].

The current study is the first to show changes in ANS function at 12 months of age in offspring of women affected by GDM. Typical changes include a lower ACC measured by PRSA. Using RR intervals instead of heart rate, the lower AAC (negative values) reflect higher heart rate variability, and thus an activation of the ANS.

In an anterior study we already detected changes of the fetal ANS [9]. Interestingly, during late gestation we found an increased ANS activity in fetuses of diabetic mothers, this interpreted as an activation of the fetal ANS. Now it seems that this over-activation might lead to a medium-term impairment of offsprings’ cardiovascular systems.

Increased sympathetic activity is associated with later development of hypertension [15,16,17,18,19,20,21]. Furthermore, there is evidence of an association between intrauterine exposure to GDM and the development of high blood pressure in later life. Our results are the first showing the link between in utero ANS activation and ANS impairment at one year of age. Further follow-up examinations are warranted to gain more insights into possible long-term cardiovascular damages.

So far, it is unclear what these results imply for children at this age and, more importantly, later in life. At least in adults, the PRSA technique has been used to demonstrate abnormalities after pulmonary vein ablations [12,14], which can be used for surveillance of growth restricted fetuses [22] or, in elderly patients as a predictor of mortality after myocardial infarction [14]. In a sheep model it was used to monitor the fetal wellbeing and for detection of fetal distress during labor [23].

Other cardiac utilities have to be used to prove that a lower ACC affects the overall performance and cardiopulmonary function.

The anthropometric and other cardiovascular data did not show any significant differences between the two groups in these children at one year of age. Published research data show a correlation between maternal GDM and arterial hypertension, and obesity in older children [4,24,25]. Nevertheless, the effect of GDM on the cardiovascular system of the offspring has not been systematically studied and understood.

Obesity in pregnancy is certainly a lifestyle problem, and without preventive strategies, families will impart it to their children, which will then have the same problem in the future. It is already known that children of former GDM pregnancies tend to have obesity which will certainly increase the risk of cardiovascular diseases. Further follow-up studies are certainly necessary to discover the possible onset in childhood. This is especially important to develop future preventive and therapeutic strategies.

A limitation of our study is the high number of subjects that were lost to follow up (39%). This is related to movements of the parents, especially to more rural areas. Thus, contact data were often missing. Another limitation was the blood pressure measurement. It would certainly have been more precise to have a 24-h blood pressure measurement. However, for ethical reasons we did not perform 24-h RR measurement in 12-month-old children for study reasons only. However, in order to minimize measurement inaccuracies, we performed three consecutive RR measurements within a defined period of time.

## 5. Conclusions

PRSA may offer the first in utero surveillance tool linking GDM pregnancy with future cardiovascular dysfunction in offspring. GDM may have a relevant adverse impact on the ANS at least at the age of 12 months. Further studies are required to determine the time when these changes occur, if they are reversible and what long-term effects they may have on the cardiovascular system and children’s health.

Therefore, pediatric cardiovascular follow-up from early childhood is essential and preventive programs have to be established for children at risk.

## Figures and Tables

**Table 1:** Anthropometric data.

Anthropometric Data	Study Group (*n* = 63)	Control Group (*n* = 62)	*p*-Value
Age (months)	12.6 ± 1.7	12.4 ± 2.2	0.71
Height (cm)	76.55 ± 3.90	76.95 ± 3.63	0.57
Percentile	61 ± 23	55 ± 24	0.17
Weight (g)	9720.70 ± 1295.19	9710.69 ± 1201.65	0.97
Percentile	47 ± 24	47 ± 26	0.96
BMI	17 ± 1	16 ± 2	0.53
Percentile	43 ± 29	47 ± 30	0.53

Data are displayed as mean ± SD; BMI, body mass index.

**Table 2:** Cardiovascular data.

(a) Blood pressure and Intima-Media Thickness.			
**Cardiovascular Data**	**Study Group (*n* = 63)**	**Control Group (*n* = 62)**	***p*-Value**
RR systolic (mm Hg)	88 ± 9	90 ± 7	0.43
RR diastolic (mm Hg)	60 ± 12	58 ± 9	0.35
Aortic IMT (cm)	0.10 ± 0.02	0.11 ± 0.02	0.52
(b) Average acceleration capacity.			
**Cardiovascular Data**	**Study Group (*n* = 48)**	**Control Group (*n* = 39)**	***p*-Value**
AAC (ms)	−20.10 ± 3.04	−18.87 ± 4.00	0.02 *

Data are displayed as mean ± SD. AAC, average acceleration capacity; RR, blood pressure measurement after Scipione Riva Rocci; IMT, intima media thickness; * statistically significant (*p* < 0.05) difference between both groups.
